# Supplementary material for: Precise radiometric age establishes Yarrabubba, Western Australia, as Earth’s oldest recognised meteorite impact structure
Source: Nat Commun. 2020 Jan 21;11:300. doi: 10.1038/s41467-019-13985-7 (PMC6974607; doi:10.1038/s41467-019-13985-7)

Precise radiometric age establishes Yarrabubba, Western Australia, as Earth's oldest recognised meteorite impact structure

Timmons M Erickson[1, 2, 3†*], Christopher L Kirkland[2‡], Nicholas E Timms[2†], Aaron J. Cavosie[2†], Thomas M Davison[4]

1. Jacobs – JETS, Astromaterials Research and Exploration Science division, NASA Johnson Space Center, 2101 NASA Parkway, Houston, TX, 77058, USA

2. The Institute for Geoscience Research (TIGeR), ([†]Space Science and Technology Centre), ([‡]Centre for Exploration Targeting – Curtin Node), School of Earth and Planetary Sciences, Curtin University, GPO Box 1984, Perth, WA, 6845, Australia

3. Center for Lunar Science and Exploration, Lunar and Planetary Institute, Universities Space Research Association, 3600 Bay Area Blvd., Houston, TX, 77058, USA

4. Impacts and Astromaterials Research Centre, Department of Earth Science and Engineering, Imperial College London, London SW7 2AZ, UK

*Corresponding Author: Timmons.M.Erickson@nasa.gov

Supplemental Table 1. U - Th - Pb results for shocked zircon from Yarrabubba monzogranite and Barlangi granophyre

Texture targeted	*Grain. spot*	^{238}U *(ppm)*	^{232}Th *(ppm)*	$^{232}Th/^{238}U$	*f204 (%)*	$^{238}U/^{206}Pb$	$\pm 1\sigma$	$^{207}Pb/^{206}Pb$	$\pm 1\sigma$	$^{238}U/^{206}Pb^*$	$\pm 1\sigma$	$^{207}Pb^*/^{206}Pb^*$	$\pm 1\sigma$	$^{238}U/^{206}Pb^*$ *date (Ma)*	$\pm 1\sigma$	$^{207}Pb^*/^{206}Pb^*$ *date (Ma)*	$\pm 1\sigma$	*Disc. (%)*	*Disc. % 2σ lim.*
Sample: 14YB07, Yarrabubba monzogranite, 680272 mE, 6991390 mS, zone 50J, GDA1984																			
Oscillatory	14YB07-z1.1	468	802	1.77	1.366	2.386	0.071	0.18031	0.00262	2.419	0.072	0.16813	0.00335	2230	58	2539	33	12.2	4.9
Oscillatory	14YB07-z12.1	449	311	0.72	0.640	2.527	0.048	0.16253	0.00106	2.543	0.048	0.15683	0.00153	2138	35	2422	17	11.7	8.6
Oscillatory	14YB07-z17.2	483	466	1.00	0.703	2.209	0.042	0.17815	0.00109	2.225	0.043	0.17188	0.00153	2393	39	2576	15	7.1	3.5
Oscillatory	14YB07-z18.1	361	285	0.82	0.656	2.127	0.043	0.17780	0.00126	2.141	0.043	0.17194	0.00169	2471	42	2577	16	4.1	
Oscillatory	14YB07-z20.1	229	219	0.99	1.372	2.151	0.048	0.18763	0.00506	2.181	0.050	0.17539	0.00718	2433	48	2610	68	6.8	
Oscillatory	14YB07-z22.1	302	278	0.95	0.695	2.005	0.042	0.17654	0.00128	2.019	0.042	0.17034	0.00175	2593	46	2561	17	-1.3	
Oscillatory	14YB07-z22.2	337	295	0.91	0.223	2.035	0.042	0.17919	0.00130	2.040	0.042	0.17721	0.00146	2572	44	2627	14	2.1	
Oscillatory	14YB07-z7.1	384	475	1.28	1.112	2.473	0.048	0.17127	0.00126	2.500	0.049	0.16136	0.00206	2169	37	2470	22	12.2	8.2
Oscillatory	14YB07-z7.2	371	342	0.95	1.093	2.297	0.046	0.17411	0.00232	2.322	0.046	0.16437	0.00285	2308	39	2501	29	7.7	1.2
Sample: 14YB03, Barlangi granophyre, 681089 mE, 6994785 mS, zone 50J, GDA 1984																			
Granular	14YB03-z1.1	1205	617	0.53	1.601	5.252	0.134	0.15175	0.00084	5.337	0.137	0.13759	0.00166	1107	27	2197	21	49.6	48.0
Granular	14YB03-z1.2	2168	779	0.37	2.225	8.834	0.284	0.15690	0.00158	9.035	0.291	0.13721	0.00236	677	21	2192	30	69.1	64.2
Granular	14YB03-z1.3	1048	806	0.79	1.374	4.944	0.191	0.15274	0.00089	5.013	0.194	0.14057	0.00163	1173	43	2234	20	47.5	45.5
Granular	14YB03-z1.4	2201	939	0.44	3.175	8.656	0.188	0.16244	0.00081	8.940	0.195	0.13437	0.00197	684	14	2156	26	68.3	64.6
Core	14YB03-z11.1	759	212	0.29	0.874	3.590	0.063	0.19375	0.00112	3.622	0.064	0.18596	0.00164	1572	25	2707	15	41.9	43.1
Rim (w. bdl)	14YB03-z13.1	1890	314	0.17	2.578	13.868	0.227	0.15635	0.00182	14.234	0.236	0.13357	0.00307	438	7	2145	40	79.6	70.5
Core	14YB03-z13.2	881	472	0.55	0.128	1.986	0.035	0.19566	0.00080	1.988	0.035	0.19453	0.00085	2626	38	2781	7	5.6	3.1
Core	14YB03-z13.3	859	430	0.52	0.453	2.531	0.044	0.19198	0.00085	2.543	0.044	0.18794	0.00106	2138	32	2724	9	21.5	21.8
Rim (w. bdl)	14YB03-z13.4	2375	813	0.35	3.244	11.202	0.182	0.16085	0.00207	11.577	0.190	0.13220	0.00322	534	9	2127	43	74.9	65.7
Core	14YB03-z15.1	3865	540	0.14	0.696	3.810	0.061	0.15385	0.00082	3.836	0.061	0.14767	0.00117	1493	22	2319	14	35.6	35.9
Granular	14YB03-z22.1	697	345	0.51	2.365	6.455	0.114	0.15586	0.00123	6.612	0.119	0.13494	0.00284	908	15	2163	37	58.0	52.2
Granular	14YB03-z5.1	834	1025	1.27	0.667	2.866	0.050	0.14855	0.00079	2.886	0.051	0.14263	0.00121	1918	30	2259	15	15.1	12.8
Granular	14YB03-z5.2	1706	682	0.41	1.317	3.578	0.142	0.14990	0.00063	3.626	0.144	0.13823	0.00243	1570	58	2205	31	28.8	23.2
Granular	14YB03-z6.1	1665	888	0.55	2.503	7.637	0.306	0.15735	0.00091	7.833	0.315	0.13522	0.00210	775	30	2167	27	64.3	60.2
Granular	14YB03-z6.2	1140	868	0.79	1.351	4.597	0.108	0.15361	0.00087	4.660	0.109	0.14163	0.00159	1253	27	2247	19	44.2	43.1
Granular	14YB03-z6.3	1186	836	0.73	1.084	3.434	0.058	0.15090	0.00072	3.472	0.059	0.14129	0.00125	1632	25	2243	15	27.3	26.4
Granular	14YB03-z6.4	2473	819	0.34	2.599	8.042	0.298	0.16167	0.00079	8.257	0.306	0.13866	0.00176	737	27	2211	22	66.7	64.0
Granular	14YB03-z9.1	665	691	1.07	0.615	3.255	0.058	0.14395	0.00089	3.275	0.059	0.13850	0.00131	1718	27	2209	16	22.2	20.5
Granular	14YB03-z9.2	902	843	0.97	0.665	3.541	0.061	0.14585	0.00084	3.564	0.062	0.13997	0.00124	1594	25	2227	15	28.4	27.6

F204% is the fraction of non-radiogenic Pb in 206Pb calculated by the 204Pb correction approach. * indicates ratios and ages corrected for common Pb. Disc (%) is percentage discordance calculated as [(207Pb/206Pb age – 238U/206Pb age)/ 207Pb/206Pb age] x 100. Disc. % 2σ lim. is the percentage discordance for the closest approach of the two sigma uncertainty ellipse to the conventional concordia curve. A null value is within two sigma analytical uncertainty of concordia.

Supplemental Table 2. U - Th - Pb results for shocked monazite from Yarrabubba monzogranite and Barlangi granophyre

Group ID	*Grain. spot*	^{238}U *(ppm)*	^{232}Th *(ppm)*	$^{232}Th/^{238}U$	*f204 (%)*	$^{238}U/^{206}Pb^*$	$\pm 1\sigma$	$^{207}Pb^*/^{206}Pb^*$	$\pm 1\sigma$	$^{238}U/^{206}Pb^*$ *date (Ma)*	$\pm 1\sigma$	$^{207}Pb^*/^{206}Pb^*$ *date (Ma)*	$\pm 1\sigma$	*Disc. (%)*	*Disc. % 2σ lim.*
Sample: 14YB07, Yarrabubba monzogranite, 680272 mE, 6991390 mS, zone 50J, GDA1984															
granular	14YB07-m3.2	97	39083	404	4.149	2.130	0.054	0.14359	0.00502	2481	52	2271	60	-9.3	
granular	14YB07-m3.4	382	40655	106	0.595	2.494	0.046	0.13958	0.00126	2173	34	2222	16	2.2	
granular	14YB07-m5.1	3543	177288	50	-0.100	2.769	0.064	0.14157	0.00175	1988	39	2246	21	11.5	6.8
twin	14YB07-m5.2	3271	170379	52	-0.077	2.376	0.056	0.16105	0.00300	2265	45	2467	31	8.2	1.0
granular	14YB07-m5.3	2351	121298	52	0.036	2.870	0.065	0.13991	0.00119	1927	38	2226	15	13.4	10.2
twin	14YB07-m5.4	2859	153226	54	0.025	2.802	0.073	0.16215	0.00069	1967	44	2478	7	20.6	19.5
granular	14YB07-m6.1	1937	78016	40	-0.049	2.396	0.039	0.14049	0.00045	2249	31	2233	5	-0.7	
granular	14YB07-m6.2	2008	78223	39	0.207	2.269	0.037	0.14013	0.00102	2354	32	2229	13	-5.6	-1.9
granular	14YB07-m6.3	2173	81041	37	0.193	2.292	0.038	0.13937	0.00046	2334	32	2219	6	-5.2	-2.4
granular	14YB07-m6.4	2099	89902	43	0.127	2.287	0.038	0.13991	0.00048	2338	32	2226	6	-5.0	-2.2
granular	14YB07-m6.5	2059	85546	42	0.399	2.393	0.046	0.13984	0.00056	2251	37	2225	7	-1.2	
granular	14YB07-m9.1	1829	57436	31	-0.050	2.350	0.039	0.13970	0.00045	2285	32	2223	6	-2.8	
granular	14YB07-m9.2	240	45011	188	1.456	2.800	0.088	0.13704	0.00216	1969	53	2190	27	10.1	3.0
granular	14YB07-m9.3	1578	48032	30	0.947	2.371	0.039	0.14101	0.00069	2268	32	2240	8	-1.3	
twin	14YB07-m9.4	1033	32147	31	0.114	2.766	0.061	0.14803	0.00070	1989	38	2323	8	14.4	12.5
host	14YB07-m9.5	1516	62232	41	0.232	2.310	0.038	0.15642	0.00058	2319	32	2417	6	4.1	1.3
Sample: 14YB03, Barlangi granophyre, 681089 mE, 6994785 mS, zone 50J, GDA 1984															
granular	14YB03-m1.1	479	24962	52	1.484	2.176	0.038	0.13810	0.00180	2438	35	2204	23	-10.6	-5.7
granular	14YB03-m1.2	508	30522	60	1.304	2.943	0.052	0.14167	0.00121	1886	28	2248	15	16.1	14.0
granular	14YB03-m1.3	670	33359	50	0.365	2.547	0.044	0.14145	0.00129	2135	31	2245	16	4.9	0.7
granular	14YB03-m1.4	466	38969	84	0.923	2.490	0.045	0.14110	0.00129	2176	33	2241	16	2.9	
granular	14YB03-m1.5	584	24013	41	0.816	2.342	0.051	0.14047	0.00095	2292	41	2233	12	-2.6	
granular	14YB03-m11.1	693	40242	58	2.226	2.733	0.047	0.14028	0.00116	2010	29	2231	14	9.9	6.9
granular	14YB03-m11.2	981	49340	50	4.198	1.987	0.033	0.13876	0.00132	2627	36	2212	17	-18.8	-16.9
host	14YB03-m12.1	633	41141	65	1.176	1.793	0.032	0.16016	0.00116	2858	41	2457	12	-16.3	-14.9
host	14YB03-m12.2	439	29307	67	2.948	1.136	0.025	0.14763	0.00121	4070	65	2319	14	-75.5	-92.9
twin	14YB03-m14.1	1694	42432	25	0.511	2.459	0.041	0.14812	0.00060	2200	30	2324	7	5.4	2.8
twin	14YB03-m14.2	1651	38000	23	0.987	2.270	0.037	0.14685	0.00066	2353	32	2310	8	-1.9	
granular	14YB03-m15.1	1088	25320	23	0.513	2.189	0.036	0.13875	0.00065	2426	33	2212	8	-9.7	-7.4
granular	14YB03-m15.2	1418	27174	19	-0.041	2.616	0.043	0.14172	0.00049	2087	29	2248	6	7.2	5.0
granular	14YB03-m15.3	1111	30231	27	0.186	2.740	0.046	0.14086	0.00060	2006	28	2238	7	10.4	8.6
granular	14YB03-m15.4	1788	37010	21	0.375	2.608	0.043	0.13917	0.00060	2092	29	2217	7	5.6	3.0
granular	14YB03-m15.5	909	28322	31	1.242	2.243	0.038	0.14089	0.00295	2376	33	2238	36	-6.2	
twin	14YB03-m5.1	964	22921	24	0.144	2.361	0.045	0.14469	0.00058	2277	36	2284	7	0.3	
granular	14YB03-m5.2	592	21598	37	0.921	2.247	0.039	0.13999	0.00095	2373	34	2227	12	-6.6	-3.1
granular	14YB03-m8.1	659	44588	68	0.666	2.220	0.039	0.13928	0.00093	2398	34	2218	12	-8.1	-4.9

F204% is the fraction of non-radiogenic Pb in 206Pb calculated by the 204Pb correction approach. * indicates ratios and ages corrected for common Pb. Disc (%) is percentage discordance calculated as [(207Pb/206Pb age – 238U/206Pb age)/ 207Pb/206Pb age] x 100. Disc. % 2σ lim. is the percentage discordance for the closest approach of the two sigma uncertainty ellipse to the conventional concordia curve. A null value is within two sigma analytical uncertainty of concordia.

Supplementary Fig. 1. Yarrabubba Monzogranite (14YB07) - zircon

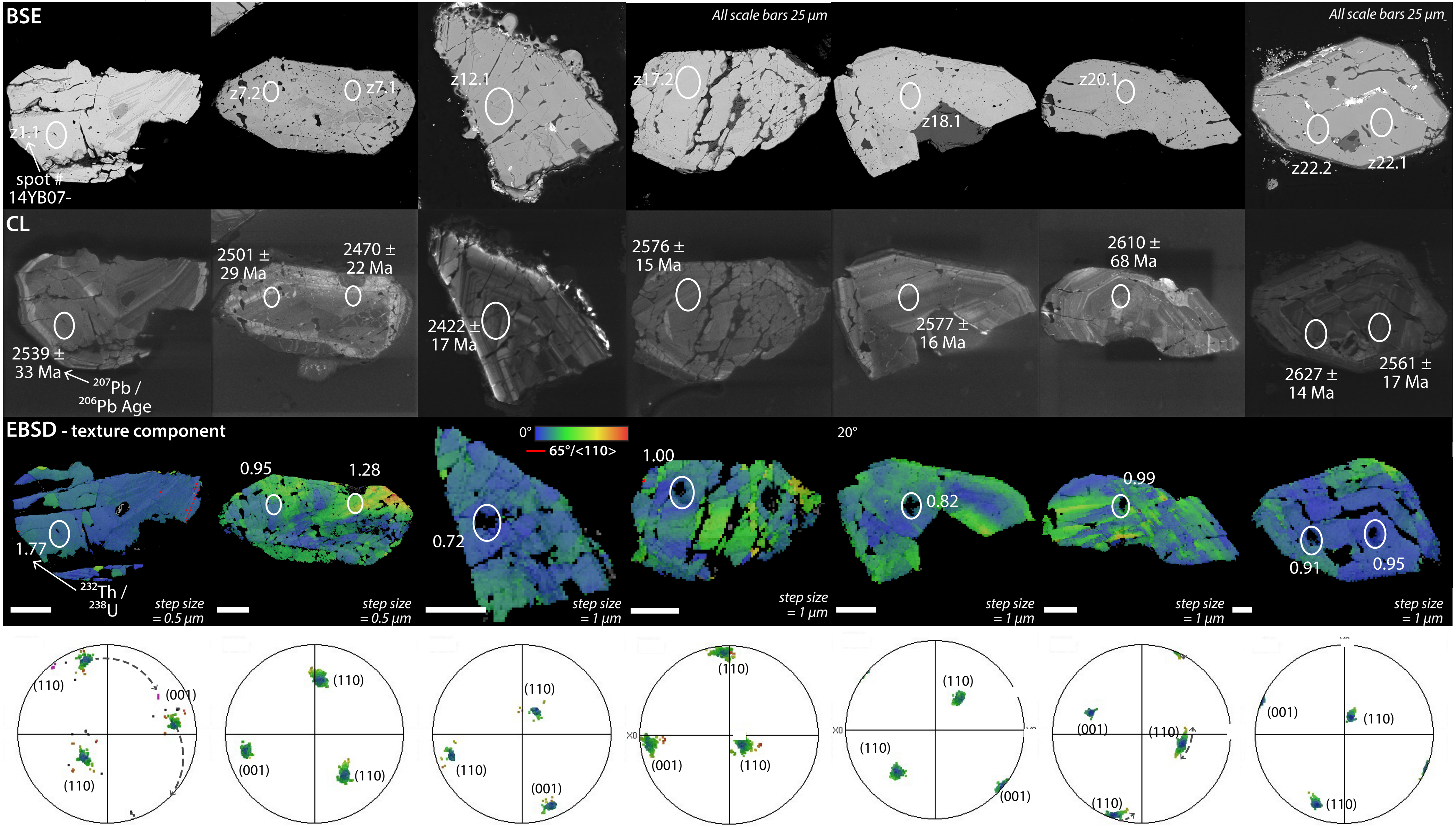

Supplementary Fig. 2. Yarrabubba Monzogranite (14YB07) - monazite

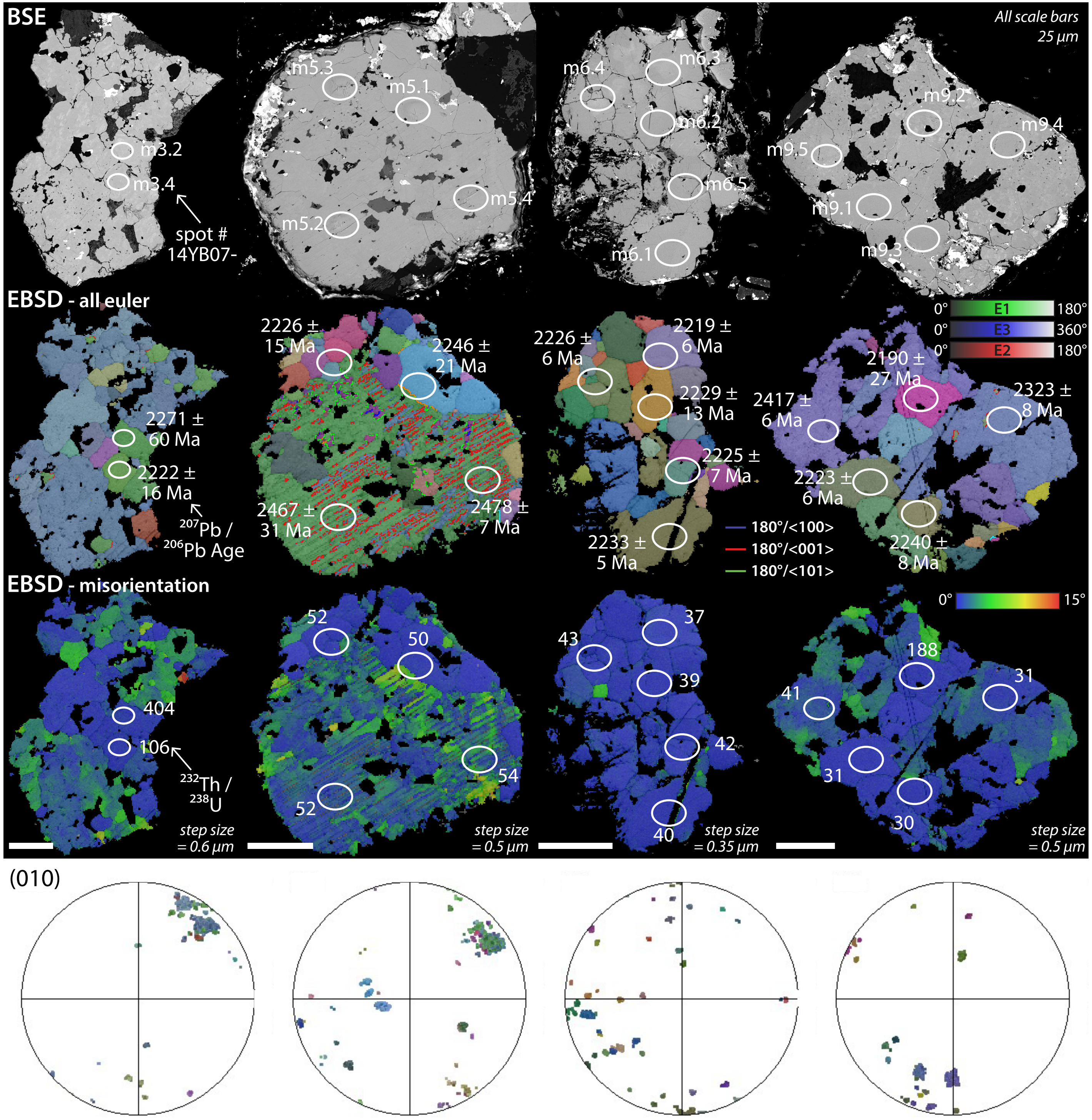

Supplementary Fig. 3. Barlangi Granophyre (14YB03) - zircon

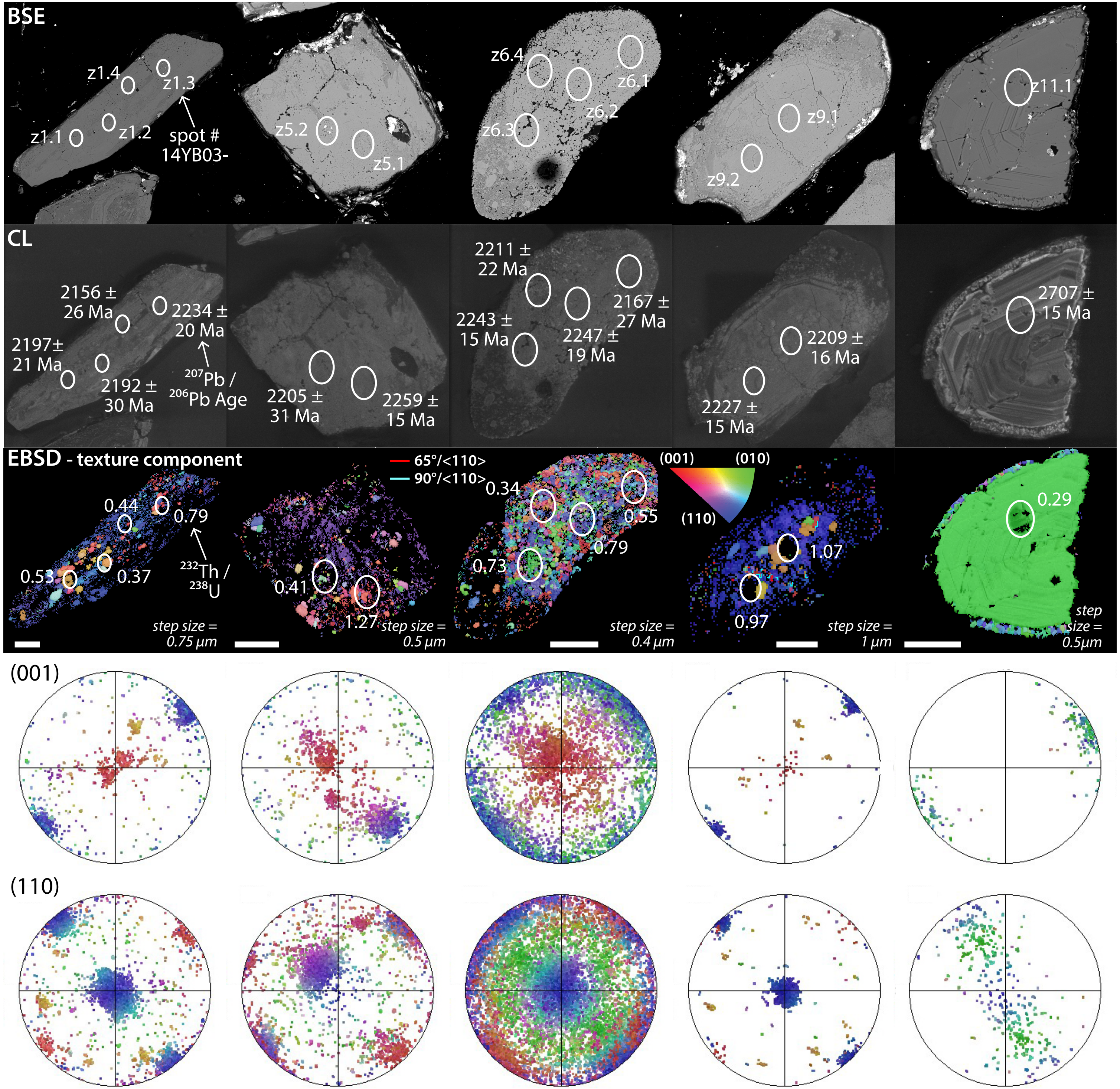

Supplementary Fig. 3 cont. Barlangi Granophyre (14YB03) - zircon

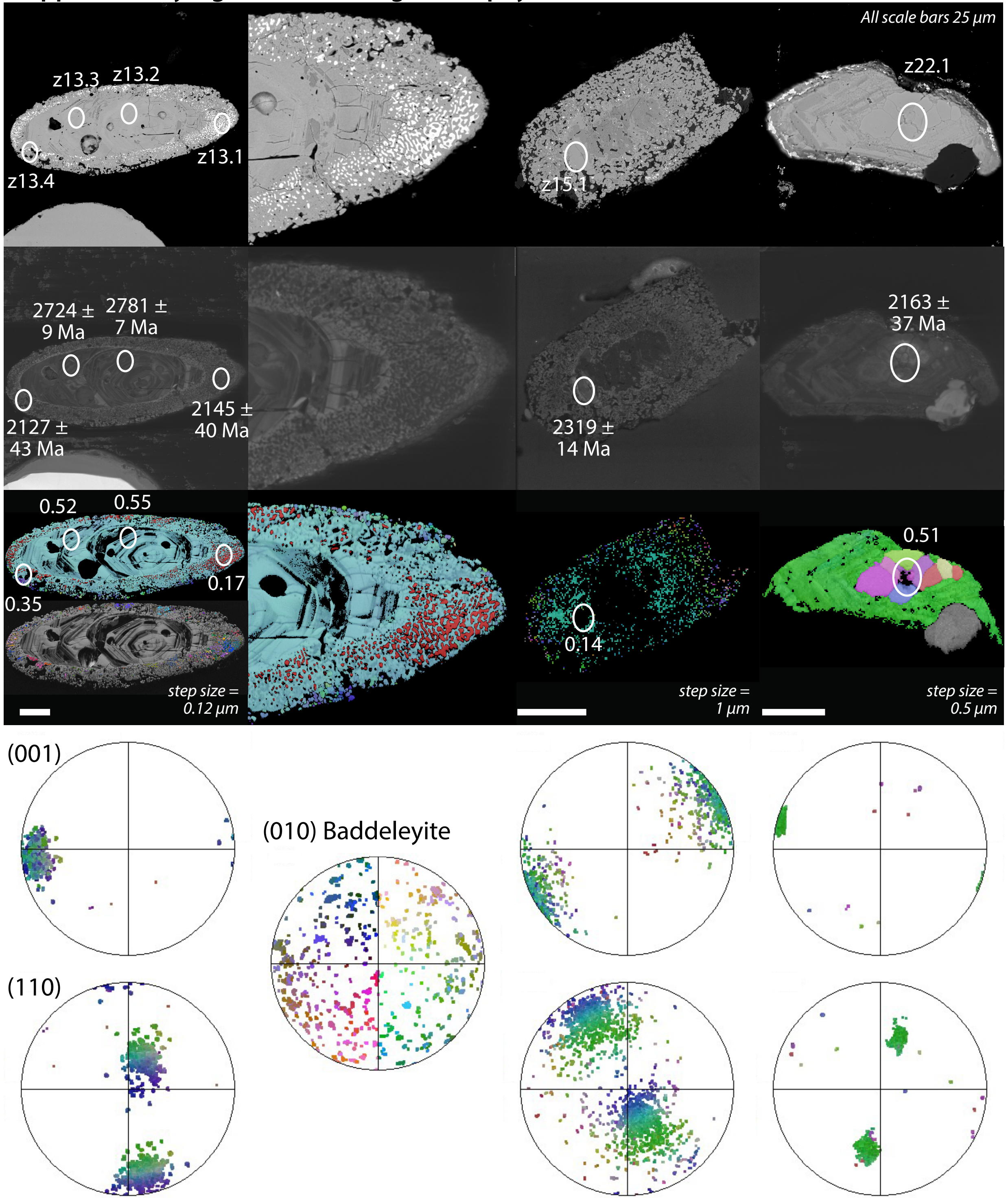

Supplementary Fig. 4. Barlangi Granophyre (14YB03) - monazite

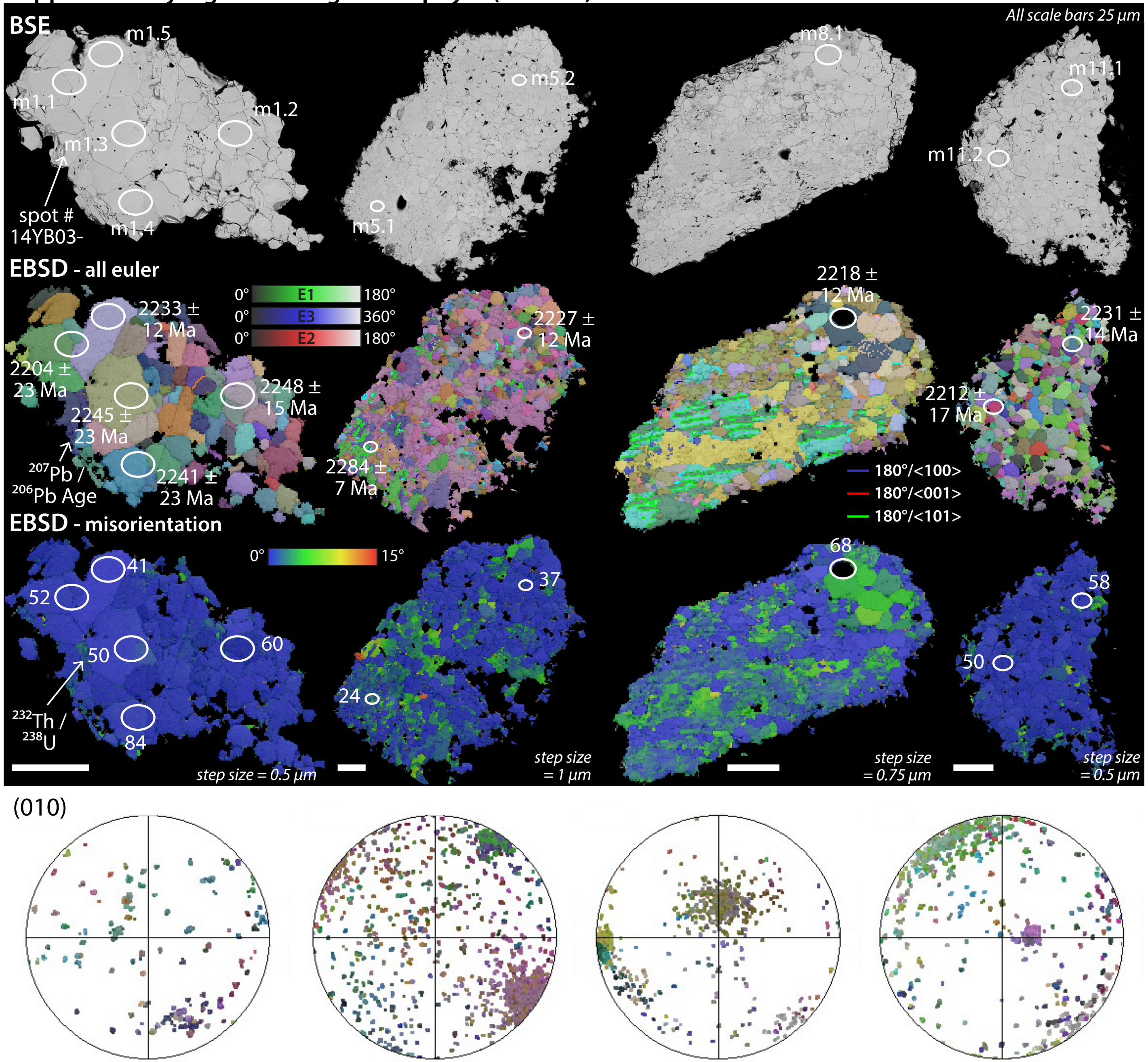

Supplementary Fig. 4 cont. Barlangi Granophyre (14YB03) - monazite

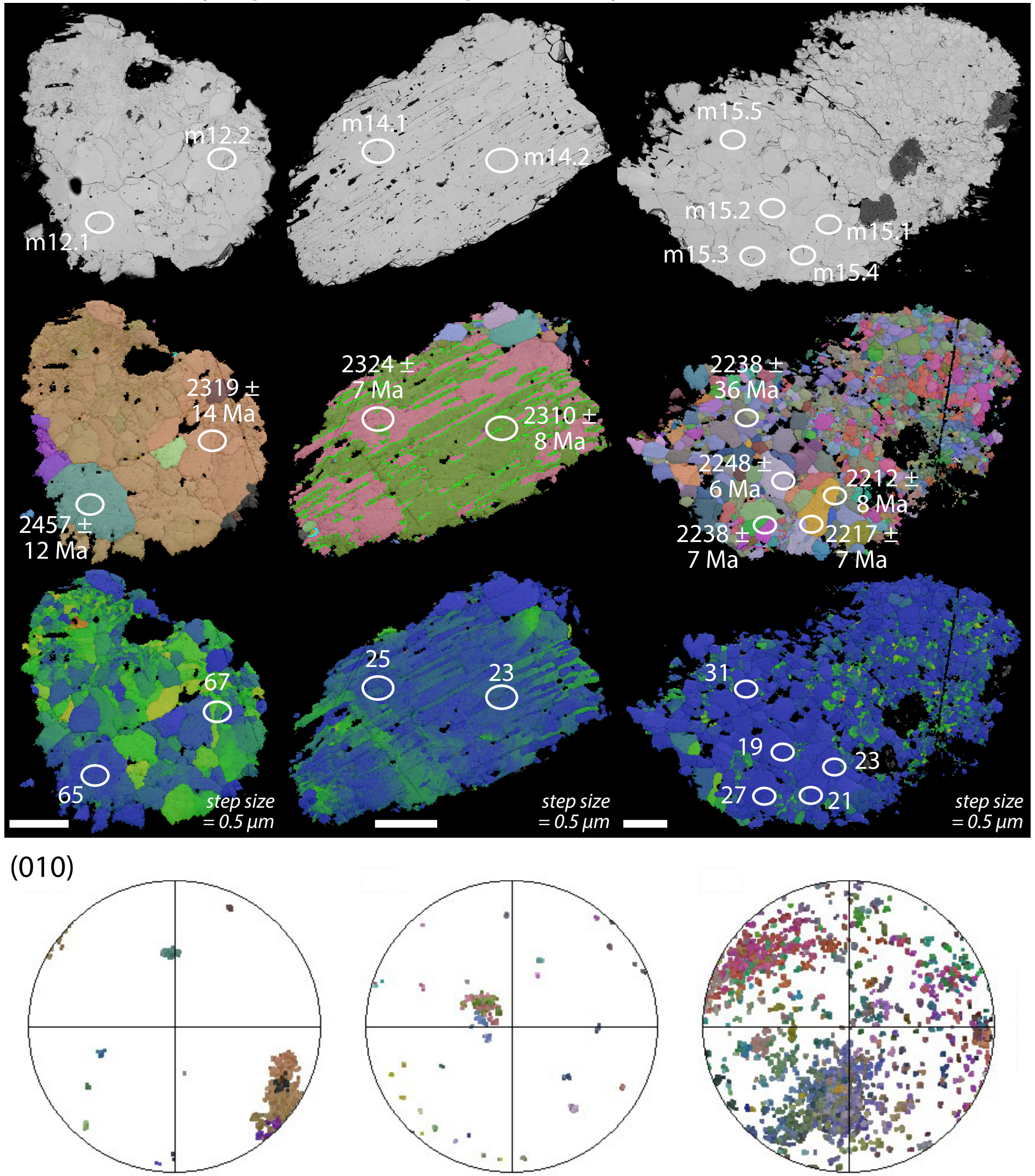

Supplement: Supplementary file 2 — Supplementary Information [file 41467_2019_13985_MOESM2_ESM.pdf]
